# Evaluation of a template for countering misinformation—Real-world Autism treatment myth debunking

**DOI:** 10.1371/journal.pone.0210746

**Published:** 2019-01-30

**Authors:** Jessica Paynter, Sarah Luskin-Saxby, Deb Keen, Kathryn Fordyce, Grace Frost, Christine Imms, Scott Miller, David Trembath, Madonna Tucker, Ullrich Ecker

**Affiliations:** 1 School of Applied Psychology/Menzies Health Institute, Griffith University, Gold Coast, Queensland, Australia; 2 Griffith Institute for Educational Research, Griffith University, Gold Coast, Queensland, Australia; 3 North West Tasmania Autism Specific Early Learning and Care Centre, St Giles Society, Burnie, Tasmania, Australia; 4 Daphne Street Autism Specific Early Learning and Care Centre, Anglicare South Australia, Adelaide, South Australia, Australia; 5 Centre for Disability & Development Research, Australian Catholic University, Melbourne, Victoria, Australia; 6 Early Intervention Services, Autism Association of Western Australia, Perth, Western Australia, Australia; 7 Menzies Health Institute Queensland, Griffith University, Gold Coast, Queensland, Australia; 8 Research and Assessment, AEIOU Foundation, Brisbane, Queensland, Australia; 9 School of Psychological Science, University of Western Australia, Perth, Western Australia, Australia; University of Wollongong, AUSTRALIA

## Abstract

Misinformation poses significant challenges to evidence-based practice. In the public health domain specifically, treatment misinformation can lead to opportunity costs or direct harm. Alas, attempts to debunk misinformation have proven sub-optimal, and have even been shown to “backfire”, including increasing misperceptions. Thus, optimized debunking strategies have been developed to more effectively combat misinformation. The aim of this study was to test these strategies in a real-world setting, targeting misinformation about autism interventions. In the context of professional development training, we randomly assigned participants to an “optimized-debunking” or a “treatment-as-usual” training condition and compared support for non-empirically-supported treatments before, after, and six weeks following completion of online training. Results demonstrated greater benefits of optimized debunking immediately after training; thus, the implemented strategies can serve as a general and flexible debunking template. However, the effect was not sustained at follow-up, highlighting the need for further research into strategies for sustained change.

## Introduction

Misinformation can have adverse consequences because misinformation-based decisions carry inherent risk of direct harm or opportunity costs. To illustrate with two public health examples: Some cancer patients choose homeopathic remedies based on misconceptions regarding proposed (but untrue) healing powers, but pay the price with higher rates of disease recurrence and death [1]. Similarly, misinformation-based rejection of vaccinations—especially in the wake of the “vaccine-autism scare” surrounding the mumps-measles-rubella vaccination—has demonstrably contributed to the resurgence of vaccine-preventable diseases [2]. Given the potentially serious implications of misinformation, we need to better understand the processes underlying the perpetuation of misinformation, and how to counter its influence [3–6].

Misinformation is resistant to corrections: people often continue to rely on corrected misinformation in their reasoning even if they understand, believe, and later remember the correction. This phenomenon has been labelled the *continued influence effect* [7–11]. Not only are corrections less effective than desired, under certain conditions poorly designed corrections can be entirely ineffective or inadvertently strengthen the misconceptions they seek to correct. Such backfire effects arise primarily in one of four cases: First, people may reject particularly authoritative corrections due to psychological reactance [12]. Second, a simple retraction that repeats the misinformation (in order to retract it) without sufficient detail can potentially create or strengthen misconceptions by making the misinformation more familiar or spreading it to new audiences—after all, publishing a statement that “it is false that the MMR vaccine causes autism” implies that someone believes, or once believed, it does [11]. Third, emotive manipulations—such as use of images of sick children when correcting vaccine myths—can backfire, potentially because of a misattribution of the evoked fear [10, 13]. Finally, if misinformation supports a strongly held worldview, a correction can be interpreted as an attack on one’s core beliefs or tribal identity and thus be ineffective or backfire [14–17]. It follows that there is an urgent need for appropriately designed, well-executed, and rigorously evaluated strategies to combat the problem of misinformation.

Misinformation is a particularly serious problem in the field of autism, to the point that autism has been labelled a “fad magnet” [18]. This is due to the plethora of treatments available with no scientific evidence, or even evidence of harm, which are often aggressively marketed using anecdotes, appeals to emotions, and celebrity endorsements [19, 20]. Surveys of parents [21] and professionals [22] have shown that ineffective treatments continue to be used despite being rejected by the scientific community, and that misinformation contributes to this problem [23]. However, efforts to bridge the “research-to-practice gap” and debunk misinformation about which treatments are effective have had limited impact [20]. This may, at least in part, be due to reliance on simple retractions and provision of factual information alone, authoritative corrections, and not considering whether corrections may be seen as an attack on core beliefs.

Recent research has proposed a number of mechanisms to improve the impact of corrections, which have yet to be systematically applied and tested in a real-world setting. The aim of this study was to test the efficacy of combining these mechanisms in an “optimized-debunking” manipulation. Our general approach was based on the notion that corrections are more effective if they do not just communicate *that* a piece of information is false (e.g., a simple retraction that a practice is not evidence-based), but also detail *why* it is false, and what led people to believe it in the first place. It has been shown that a careful dissection of incorrect arguments can help promote truth, and that detailed refutations are more effective than plain, stripped-down retractions or the provision of factual information alone [3, 24–26]. A powerful correction ideally places emphasis on detailing facts and evidence support of them. This is especially important if a piece of misinformation carries a specific function in a person’s mental model of an event or causality [8]. For example, if a person falsely believes in an autism epidemic brought about by vaccinations, then it is crucial to refute the misinformation *and* to concurrently provide alternative information to fill the “gap” created by the correction—in this example, that the observed rise in autism rates is mostly due to broadened diagnostic criteria and heightened awareness of the condition [27]. Moreover, it is important to design refutations that use simple language to facilitate understanding, and an empathetic, non-confrontational tone [9].

We combined this basic refutational approach with six specific, additional elements thought to boost the effectiveness of a correction [7, 9]. We briefly review these elements, in the order they were incorporated into our optimized-debunking materials, below:

1. *Source credibility—*corrections are more effective if they come from a person or institution that is high in perceived credibility. The primary driver of this effect appears to be the source’s perceived trustworthiness rather than expertise [28].
2. *Self-affirmation* interventions have been shown to make potentially worldview-inconsistent corrections “easier to swallow”—affirming a person’s values makes them more open to worldview-inconsistent information, presumably by fostering resilience to the inherent identity threat [29, 30].
3. *Social norming*—if either an injunctive or a descriptive norm is presented in support of a correction, it should facilitate acceptance of corrective information due to people’s aversion to social extremeness and the associated fear of social exclusion [31].
4. *Warning* people before exposing them to misinformation puts them cognitively on guard and may prevent them from initially believing the misinformation upon exposure, thus obviating the need for retrospective re-evaluation when receiving the correction. Warnings may also boost strategic monitoring and recollection processes that can avert reliance on misinformation even when it is activated by relevant cues at a later time [8].
5. *Graphical representations* can boost corrective impact by attracting attention, facilitating information processing and retention, and quantifying or disambiguating the corrective evidence, thus reducing the recipient’s ability to counter-argue inconvenient information [10, 30, 32, 33].
6. *Salience* of the core corrective message can enhance its effectiveness, presumably based on a link between enhanced fluency of processing and information impact [11].

Debunking materials were designed along these guidelines for use in a real-world setting. Specifically, we aimed to educate participants regarding the lack of evidence for three common ineffective autism treatments and prevent them from using and promoting these ineffective treatments. We conducted the study with early-intervention professionals because misinformation is especially problematic when disseminated by professionals who play a vital role in the translation of research to practice, and are a valued and trusted source of information for both families and other professionals [23]. We presented the optimized-debunking materials as part of a professional development intervention, and contrasted the materials’ efficacy with the impact of control training materials commonly used with this group. The main research question was: “Does optimized debunking decrease support for treatments that lack empirical evidence relative to a control intervention?” Secondary research questions were: “Are any beneficial effects of the debunking sustained over time?” and “Does the optimized-debunking intervention have any impact on participants’ support of evidence-based treatments?” Finally, we explored whether the optimized-debunking intervention and its intended purpose would be received by participants as socially valid (i.e., important and acceptable), and whether the effectiveness of the debunking might relate to participants’ deference to scientific authority and/or pre-existing attitudes towards evidence-based practice [13, 32].

## Method

### Design

The study used a 2 × 3 mixed factorial design, with the between-subjects factor condition (optimized debunking vs. control) and the within-subjects factor time (time 1, pre-intervention; time 2, post-intervention; time 3, delayed follow-up).

### Participants

Participants were recruited from four different autism early intervention centers from the Autism Specific Early Learning and Care Centres (ASELCC) across four organisations, and four states of Australia. Participants included teachers, allied health and early learning paraprofessionals working with pre-school children with autism (aged 2–6 years). Overall, 86 staff participated (*n* = 71 at time 1; *n* = 71 at time 2; *n* = 66 at time 3); *N* = 47 participants provided measures for the first two time-points and were used for the main analyses (*n* = 42 from this completed the third time point and were included for follow-up analysis). Of these 47, 44 were female, with an approximate mean age of 34 years (based on age-bracket midpoints). The average time period working with children with autism was *M* = 4.52 years, *SD* = 3.67. Participants were mostly early learning paraprofessionals such as qualified childcare staff (63.28%), followed by speech pathologists (10.6%), teachers (8.5%), occupational therapists (6.3%), behavior analysts (4.3%), and other professionals such as social workers (6.4%). Participants were matched across sessions through an anonymous, individually-generated code.

### Materials

All materials are provided in the S1 File (Online Supplement).

#### Intervention materials

Training materials in both conditions focused on three non-empirically-supported treatments (non-ESTs); namely facilitated communication, auditory integration training, and special diets, which were paired with three empirically-supported treatments (ESTs); Picture Exchange Communications System (PECS), antecedent-based intervention, and exercise [34]. The materials in the control condition were created in consultation with ASELCC staff not involved in data collection, using freely available training materials they reported were commonly used, including information from the Raising Children Network [35] and Positive Partnerships [36]. As is typical of such materials, the descriptions of demonstrably ineffective treatments included equivocal phrases such as *“Some studies have found that it* [a gluten and casein free diet] *is helpful*, *but the research had problems like low numbers of children in the study*. *Some well-designed studies have found few benefits*.*”* [35, 36]. Moreover, some of the materials may be interpreted as implying that demonstrably ineffective treatments *may* be effective, such as *“A small number of people on the spectrum who start communicating using FC* [facilitated communication] *go on to use typing without help*. *It would be helpful to know more about these people and how they became independent…”* [36]. While this statement does not explicitly state a causal relation between the treatment and the behavior change, and implicitly calls for research to identify alternative explanations for these rare cases of behavior change (e.g., the person had the communication skills but had simply not been given an opportunity to communicate previously), the statement is arguably misinterpreted easily.

The debunking materials were designed based on the refutational approach outlined earlier. The core debunking of each non-EST followed a specific structure: The non-EST was briefly introduced, while empathetically explaining why people might want to believe that the specific treatment works, but immediately discrediting the treatment’s alleged mechanism. This was followed by presentation of the principal fact to debunk the myth, namely that high-quality research has clearly shown the treatment to be ineffective. Then, the evidence from that research was summarized, and a superior alternative treatment was suggested and its treatment mechanism explained. Thus, the core debunking clarified that a given treatment is ineffective, why it is ineffective, why people might want to falsely believe it to be effective, and what an alternative treatment might be to address a particular issue or symptom.

To prepare participants for the core non-EST debunkings, these were prefaced by a more general section that highlighted how the apparent evidence for ineffective practices is often flawed because it relies on anecdotal experience and an associated illusion of causality [37, 38]. In this section, participants were also educated about the need for well-designed studies such as randomized control trials, and it was explained in a general manner why people might want to believe in the effectiveness of non-ESTs, while pointing out the harm associated with wasted resources, the creation of false hope, and potential side effects.

This general approach was supplemented by incorporating segments designed to implement the six additional facilitative factors reviewed in the Introduction: (1) The debunking materials first aimed to establish *source credibility* and build trust by emphasizing that the intervention’s motivation was a deep-rooted care for clients and a desire to assist professionals in their decision making, not authoritarian enlightenment or vested interests. We added a portrait image of the lead researcher that was pre-rated in a pilot test as high in trustworthiness (with *M* = 7.08 on 0–10 scale [*SD* = 2.14]; *N* = 25).

(2) This was followed by a mild *self-affirmation* intervention, which involved thanking the participants and making them aware that their participation demonstrated professional values and a commitment to high-quality care.

(3) We applied two *social-norming* interventions. The first was a general descriptive norm, emphasizing the strong agreement amongst professional health-care providers that intervention recommendations should be based on the best available evidence. This was later reinforced by providing an injunctive norm—namely that recommending only the treatments that work is the right thing to do—and also repeating the descriptive norm.

(4) Before exposing participants to specific misinformation regarding a non-EST, they received a simple *warning*, namely a statement that they were about to receive information about a treatment that research has shown to be ineffective. The label “Myth:” preceded introduction of non-EST misinformation.

(5) *Graphical representations*—specifically, intuitive, color-coded pie charts—were used to illustrate the evidence against non-ESTs (as well as the evidence for one selected EST). These charts specified the number of studies finding the respective treatment to be ineffective (coded in red), effective (green), or yielding inconclusive evidence (yellow). Additionally, a photographic image showing the application of an alternative EST was provided with its description.

(6) Finally, the *salience* of the core messages—the statement that a given non-EST is ineffective, the statement regarding the evidence against the treatment, and the introduction of the alternative EST—was enhanced by presenting the statements in bold black font or in colored boxes in a larger, white font.

#### Dependent measures

To assess participants’ support of the six treatments, a modified version (see S1 File) of the Early Intervention Practices Scale Revised was used (EIPS-R) [22]; note that some EIPS-R items were irrelevant for the current investigation, and were obtained for separate research into general professional beliefs and behaviors, along with a range of other measures. For this study, three EIPS-R ratings were selected *a priori* to create a composite score, calculated separately for non-ESTs and ESTs. The selected ratings were (1) a rating of the evidence base for a given treatment (rated on a scale from 0 [ineffective] to 4 [established]); (2) a rating of intended future use of a given treatment (from 0 [never] to 4 [frequently]); and (3) a rating of the likelihood of recommending a given treatment to parents (from -3 [will not recommend with high confidence] to +3 [will recommend with high confidence]). Thus, each composite score (for ESTs and non-ESTs, respectively) was calculated from 9 values (i.e., 3 ratings × 3 treatments); rating scores were transformed onto a common 0–1 scale before averaging. The non-EST support composite score was the main dependent variable of interest and showed good internal consistency at each time-point (T1, Cronbach’s α = .87; T2, α = .87; T3, α = .86). The composite score for ESTs showed adequate (T1, α = .69; T2, α = .77) to good (T3, α = .83) internal consistency at each time-point.

To assess participants’ attitudes towards evidence-based practice, we used the openness and divergence sub-scales of the Evidence-Based Practice Attitude Scale (EBPAS) [39]. Each sub-scale comprised four items (an example openness item was “*I like to use new types of therapy/interventions to help my clients*”; an example divergence item was “*I know better than academic researchers how to care for my clients*”); participants rated their level of agreement with each item on a five-point scale ranging from 0 (not at all) to 4 (to a very great extent). EBPAS openness showed good internal consistency (α = .81). However, EBPAS divergence showed poor internal consistency (α = .40); further analysis showed removal of two items led to adequate reliability (α = .69) for a two-item version (“*Clinical experience is more important than using manualised therapy/interventions”* and *“Research-based treatments/interventions are not clinically useful”*). Thus, this two-item scale was used as a divergence measure in the present study.

To assess participants’ deference to scientific authority, a modified version of the Deference to Scientific Authority Scale (DSAS) [40] was used. It included four items (e.g., “*Researchers know what is good for the public*”), and participants rated their level of agreement with each item on a six-point scale ranging from 0 (strongly disagree) to 5 (strongly agree); the scale showed adequate reliability (α = .70).

To assess the perceived social validity of our intervention, we administered a modified version (see S1 File) of the Intervention Rating Profile (IRP-15) [41]. We included six items (e.g., “*The online training was an acceptable way to improve my knowledge of autism spectrum disorder interventions*”), and participants rated their level of agreement with each item on a six-point scale ranging from 1 (strongly disagree) to 6 (strongly agree). Items were averaged for an overall score that showed excellent reliability (α = .96).

A manipulation check (see S1 File) with three questions (e.g., “*The training materials I just viewed included charts showing the evidence relating to the interventions being discussed*”) was included to confirm participants observed important differences between the control and debunking conditions. Participants rated their level of agreement with each item on a five-point scale ranging from 1 (strongly disagree) to 5 (strongly agree).

### Procedure

At time 1, participants completed the EIPS-R, EBPAS, and DSAS questionnaires. At time 2, participants were randomly assigned to either the control group or the debunking group and received the associated intervention materials, with the three non-ESTs (and their associated ESTs) presented in a random order. Participants then received the manipulation check and again completed the EIPS-R, followed by the IRP-15 scale. At time 3, participants only completed the EIPS-R. The mean interval between time 1 and time 2 was *M* = 11.23 weeks (*SD* = 2.46); the mean interval between time 2 and time 3 was approximately six weeks (control, *M* = 6.26, *SD* = 2.16; debunking, *M* = 6.33, *SD* = 2.28). The experimental surveys were administered using Qualtrics software (Qualtrics, Provo, UT). Data were collected between June and December 2017. The study was conducted with approval from the Human Research Ethics Committee of Griffith University (approval number 2017/007) with reciprocal approvals from the Australian Catholic University and the University of Tasmania, as well as gatekeeper approvals from the ASELCCs. All participants provided written informed consent via an online process (review of an information sheet and selection of agree on an online consent form), with this process approved by the ethics committee.

## Results

### Data screening

Data were screened for missing data and assumptions for analyses; the only violations were the assumption of homogeneity of variances for two *t*-tests, with Welch’s *t*-tests consequently used. For full details please see S2 File.

### Manipulation check

Each item was rated higher in the debunking compared to the control group, with a large effect supporting the effectiveness of the manipulation, see Table 1.

**Table 1:** Manipulation check.

	Group		Statistic					
	Control	Debunking	*t*	*df*	*p*	*d*	*95% CI*	
**Manipulation check**	(*n* = 26)	(*n* = 31)					*Lower*	*Upper*
1. Included charts*	1.69 (1.35)	4.48 (.85)	9.13	40.69	< .001	2.07	-3.41	-2.17
2. Gave alternative options*	2.38 (1.41)	4.35 (.798)	6.30	37.84	< .001	1.72	-2.60	-1.34
3. Professional organizations advise against	3.04 (1.15)	4.39 (.80)	5.20	55	< .001	1.36	-1.87	-.83

* *Note*. Levene’s test *p <* .05, thus equal variances not assumed and Welch’s *t*-test reported; 95% CI = 95% confidence interval of the difference

### Impact of debunking on support for non-ESTs

**Fig 1:**
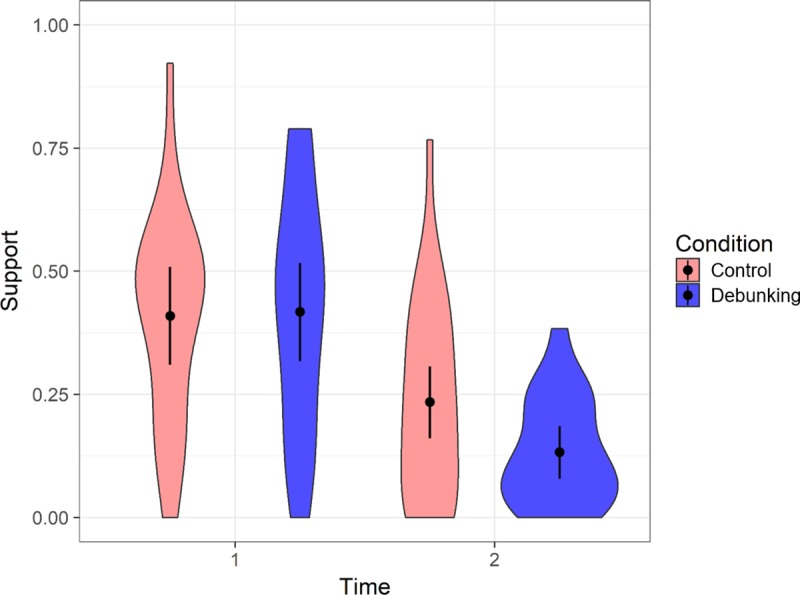
Violin plot, showing mean support for non-empirically supported treatments across control and debunking conditions at time points 1 (pre-intervention) and 2 (post-intervention). Error bars show 95% Cousineau-Morey confidence intervals (calculated following Baguley, 2012) [42]; density of score distribution is displayed using shaded areas with wider sections indicating more frequent scores.

Mean support for non-ESTs across control and debunking conditions pre- and post-manipulation is shown in Fig 1. A within-between ANOVA on support scores showed that there were large effects of time in both the control group, *F*(1, 19) = 7.98, *p =* .01, partial η^2^ = .30, and the debunking group, *F*(1, 26) = 49.28, *p* < .001, partial η^2^ = .66. While this indicated that both interventions led to a decline in non-EST support, the relevant test of our main hypothesis concerned the time × condition interaction, which was significant with a large effect, *F*(1, 45) = 11.13, *p* = .002, partial η^2^ = .20. This interaction showed that the optimized-debunking intervention was more effective at reducing non-EST support than the standard control intervention. At time 1, conditions did not differ significantly, *F* < 1. At time 2, the debunking group showed significantly lower non-EST support than the control group, with a large effect, *F*(1, 45) = 10.96, *p =* .002, partial η^2^ = .20.

### Impact of debunking on support for ESTs

**Fig 2:**
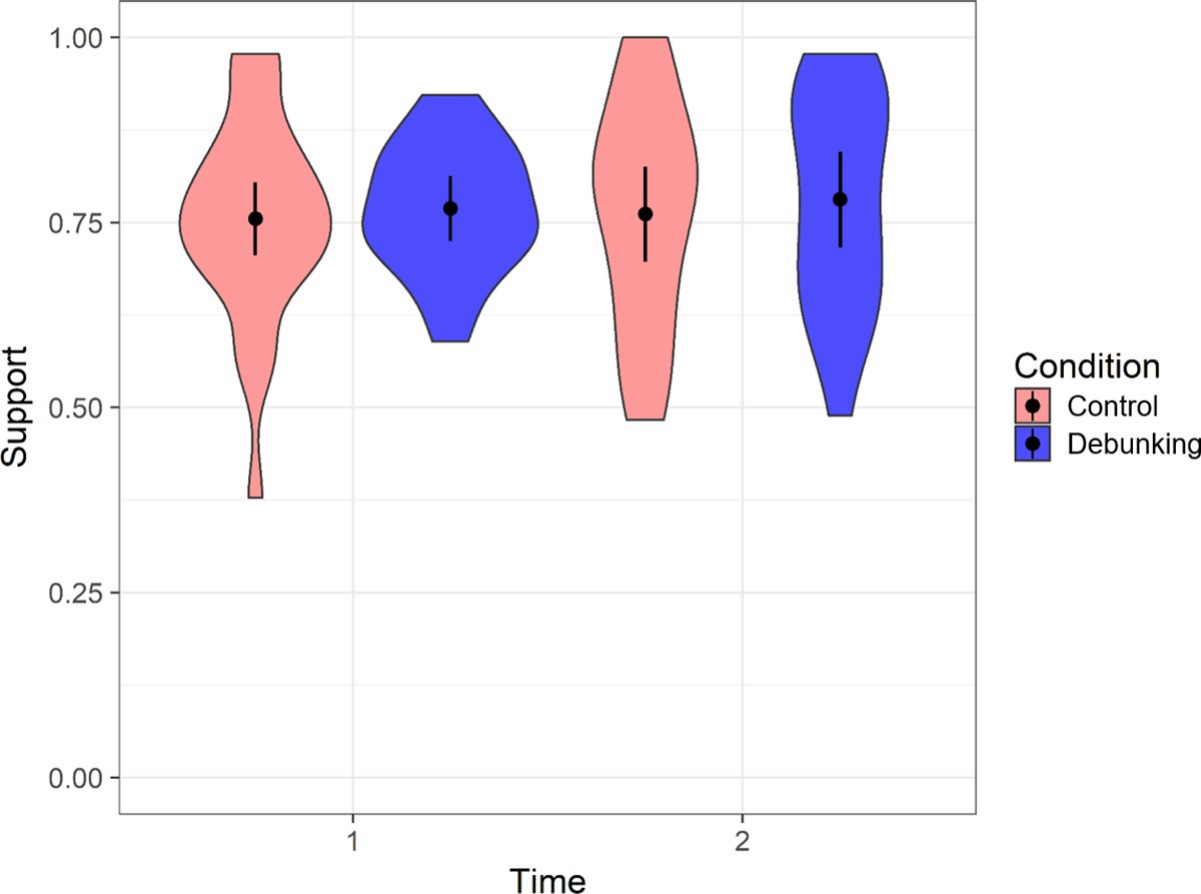
Violin plot, showing mean support for empirically supported treatments across control and debunking conditions at time points 1 (pre-intervention) and 2 (post-intervention). Error bars show 95% Cousineau-Morey confidence intervals (calculated following Baguley, 2012) [42]; density of score distribution is displayed using shaded areas with wider sections indicating more frequent scores.

Mean support for ESTs across control and debunking conditions pre- and post-manipulation is shown in Fig 2. There were no significant simple main effects of time (control, *F* < 1; debunking, *F*(1, 26) = 1.78, *p* = .194, partial η^2^ = .06), or condition (*F* < 1 for times 1 and 2). The interaction of time × condition was not significant, *F*(1, 45) = 2.35, *p* = .13, partial η^2^ = .05.

### Impact of optimized debunking over time

At time 3, groups did not differ significantly regarding their support of non-ESTs, *F* < 1. They also did not differ significantly in their support of ESTs, *F*[1, 40] = 3.62, *p* = .06, partial η^2^ *=* .08.

### Social validity

Participants from the control (*M* = 3.92, *SD* = 1.42) and debunking condition (*M* = 4.05, *SD* = 1.26) rated the social validity of the materials as moderately high (on a 1–6 scale); ratings did not differ significantly, *t* < 1.

### Links between attitudes and practice support

We calculated correlations between support change (from time 1 to times 2 and 3, respectively) and attitude measures (see Table 2). Applying α = .002 to account for multiple comparisons, none of the correlations were significant. Numerically, however, the correlation between non-EST support change from time 1 to time 3 and the EBPAS openness score was large, suggesting greater openness (to using manualized or new practices) is negatively related to sustained effectiveness of debunkings over time.

**Table 2:** Correlations between attitude measures and change in support for non-ESTs and ESTs.

	Deference to Scientific Authority		EBPAS Divergence		EBPAS Openness	
	Control (*n* = 19)	Debunking (*n* = 23)	Control (*n* = 19)	Debunking (*n* = 23)	Control (*n* = 19)	Debunking (*n* = 23)
Non-EST						
Δ T1/T2	.08	-0.17	.10	-.17	.36	-.20
Δ T1/T3	.20	-.27	-.14	-.17	.22	-.52
EST						
Δ T1/T2	.12	.004	.07	-.38	-.005	.001
Δ T1/T3	-.12	-.004	-.07	.38	.005	-.001

*Note*. Δ T1/T2 and Δ T1/T3 refer to support change from time 1 to time 2 and time 3, respectively; EBPAS, Evidence-Based Practice Attitude Scale

## Discussion

In this study, we designed an optimized-debunking intervention based on recommendations from the cognitive science literature [7, 9], systematically implementing a set of generalizable principles. We trialed this approach in an area that has been highly susceptible to misinformation, namely autism treatment. We demonstrated that an optimized-debunking intervention was more effective than a treatment-as-usual intervention at reducing support for non-empirically-supported treatments. Our approach has potential to serve as a flexible template for both real-world application and future research. Our findings expand significantly previous work in this area, which has used debunking materials created less systematically and/or with fewer elements incorporated. Our research confirmed the positive effects of weight-of-evidence information and graphical representations, while avoiding backfire effects potentially arising from emotive or confrontational debunkings [30, 32].

The finding that a refutation was initially successful, but its effect was not sustained over time, is consistent with the findings of Swire et al [26]. Swire et al. argued that after a delay, refuted “myths” can again be falsely accepted as true because recollection for the details of the refutation fades over time, while the myth’s familiarity—potentially boosted by the refutation itself—remains high. This may be particularly important in the field of autism where media, celebrity endorsement, as well as endorsement of fad treatments by professionals is common [19], and the myths may therefore be frequently encountered. This highlights the need for future research into repeated and varied refutational interventions to achieve long-term belief change.

We explored the potential association between attitudes and post-debunking support changes. The relationships were all non-significant, including the relationship between support change and deference to scientific authority, which was identified as a predictor in previous research [32]. Yet, there was a tendency for openness toward new interventions to be negatively linked to reductions in non-EST support; this is consistent with Paynter et al. [23] who similarly found that openness was linked to greater use of non-ESTs. Speculatively, openness may make a practitioner more vulnerable to the promotion of non-ESTs through misinformation, and thus effectively more resistant to science-based corrections. This notion requires further investigation and highlights the need to promote healthy skepticism.

While results provide promising support for the optimized-debunking principles employed, limitations are acknowledged. First, attrition or non-completion of one or more components occurred; this may have been due to the anonymous nature of participation, and anecdotally was linked to changes in staffing, availability of staff, and ability of staff to dedicate time to the activity. Future research could use individualized sign-ins and appropriate incentives (e.g., professional development certificates), to ensure better retention. Second, we cannot ascertain which components of our intervention were the “active ingredients.” As such, there is a need for component analysis in future research to evaluate which element/combination of elements is required for successful debunking, especially under conditions where the correction is attitude-dissonant. Finally, our outcome measure was support for non-ESTs rather than actual non-EST use. While this is in line with almost all investigations of debunking [13, 32], there is a clear need for future research to also investigate debunking effects on actual behavior. However, at least one study [23] has found significant links between attitudes toward non-ESTs and self-reported use.

To conclude, this study introduced an optimized-debunking template, and provided promising evidence for its utility. Given the significant public health impact of misinformation, development of effective debunking strategies is as vital as promoting effective evidence-based interventions.

## Supporting information

S1 File Supplemental material. (DOCX)

S2 File Data screening. (DOCX)

S3 File Dataset. (SAV)
