# Supplementary material for: Evaluation of a template for countering misinformation—Real-world Autism treatment myth debunking
Source: PLoS One. 2019 Jan 30;14(1):e0210746. doi: 10.1371/journal.pone.0210746 (PMC6353548; doi:10.1371/journal.pone.0210746)
Supplement: S1 File — (DOCX) [file pone.0210746.s001.docx]

**Online Supplement: Materials and Measures**

**Online Supplement A:** Training Materials

**Online Supplement B:** Intervention Practices Scale

**Online Supplement C:** Manipulation Check

**Online Supplement D:** Social Validity Scale

# Control Training Materials

Fact Sheet - Auditory Integration Training (AIT)

**What is Auditory Integration Training (AIT)?**

Auditory Integration Training (AIT) is a type of sound therapy, similar to the Tomatis method. It aims to reduce sensitivity to sounds or other problems with how sounds are processed.

**Who is Auditory Integration Training (AIT) for?**

Auditory Integration Training (AIT) can be used for children with autism spectrum disorder (ASD), aged three years or older, who have additional sensory problems like painful or hypersensitive hearing.

It isn’t suitable for children under three years, or children with an ear wax problem, inner ear damage, ear infections or hearing loss.

**What is Auditory Integration Training (AIT) used for?**

Auditory Integration Training (AIT) aims to reduce:

·         distortions in hearing

·         extremely sensitive hearing

·         irregularities in how sounds are processed.

These difficulties can cause discomfort or confusion in children with autism spectrum disorder (ASD).

Some practitioners also claim that AIT can help to improve speech and language difficulties and other core features of ASD.

**Where does Auditory Integration Training (AIT) come from?**

Auditory Integration Training (AIT) was developed in the 1960s by an ear, nose and throat specialist, Dr Guy Berard, with the aim of reducing the effects of auditory damage. AIT was first used for people with autism spectrum disorder (ASD) in 1975.

**What is the idea behind Auditory Integration Training (AIT)?**

Auditory Integration Training (AIT) is based on the idea that our behaviour can be influenced by how we hear. It’s also believed that hypersensitive hearing can limit people’s ability to learn and pay attention. The therapy aims to reduce sensitivity to sounds and also other problems with how sounds are processed.

**What does Auditory Integration Training (AIT) involve?**

Children attend two 30-minute training sessions a day for 10 days. In each session, children listen to music on headphones. The music has been altered to remove certain sounds, and the volume is carefully controlled.

The therapy starts by presenting familiar sounds. Over time, more challenging sounds (usually those with a high or low frequency) are introduced. This helps children slowly get used to the sounds so they’re no longer a problem.

**Does Auditory Integration Training (AIT) work?**

There’s no evidence that Auditory Integration Therapy (AIT) or other sound therapies work as treatments for autism spectrum disorder (ASD). There’s no evidence that AIT helps with speech and language or the core features of ASD.

It’s worth noting that the link between sensitive hearing and ASD isn’t completely clear and that auditory therapies are still in early development.

To reduce or prevent other hearing issues, it’s recommended that children taking part in AIT are examined at the beginning, middle and end of the AIT therapy by a qualified health care professional or auditory specialist. This will help avoid problems like ear wax or fluid build-up and possible damage to eardrums.

Fact Sheet - Gluten-free, casein-free diet

[Picture]

**What is the issue?**

Many families try ‘alternative’ ways to help their child on the autism spectrum. One thing that some parents try is the ‘gluten free, casein free diet’. This means not eating food made from wheat (like bread, pasta and cake) or dairy (like milk, cheese and yoghurt). There has been a lot written about this kind of diet.

**Why do people try this?**

There are a few different ideas behind the diet. One is that these types of foods might not be broken down in the stomach properly and that some of the molecules can get from the stomach to the bloodstream. Some people think that this can have an effect on the way the brain functions.

**What does the research say?**

Research about this diet has found different results. There haven’t been many good quality studies about how the diet changes the behaviours of autism. Some studies have found that it is helpful, but the research had problems like low numbers of children in the study. Some well-designed studies have found few benefits.

A review article written in 2013 looked at a number of research studies over the last few years. This article reported that there were problems with the way a lot of the research was conducted. Some of the studies didn’t look at the effects of the diet. In about half the studies, the children were involved in other therapy so it is hard to know if it was definitely the diet that helped them. Most of the studies did not look at the impact on the families’ quality of life. This review said that at this stage, there is little scientific evidence to say that a gluten free, casein free diet is helpful for individuals on the spectrum.

**In summary**

High quality studies have not shown a link between the diet and an improvement in autism.

Fact Sheet - Facilitated communication & autism

[Picture]

**What is the issue?**

Facilitated Communication (FC) is a way of helping people who can’t talk to communicate. In FC, a helper called a ‘facilitator’ helps the person to touch or point to letters on a board or keyboard. The facilitator holds the person’s hand, wrist, arm or other body part while the person types. The facilitator’s job is to help the person slow down, to make them more stable or to help them pull back from the keyboard before they type the next letter.

FC was first used with people with cerebral palsy in the 1970s. It started to be used with people on the autism spectrum in the 1990s. Reports about FC often say that the individuals have very good reading and writing skills when they use FC, even when these skills have never been seen before.

FC is argued about because it is hard to know who is really typing the messages: Is it the person with the disability or the person helping them? There has been a lot of research about FC to try to find out who is communicating. It is important to know if the messages made using FC are really coming from the person with the disability.

**What is the idea behind FC?**

Supporters of FC think that the communication problems seen in autism are to do with problems with movement, not cognitive or social difficulties. The idea is that by supporting the person’s hand or other body part, the person will be better able to type what they want to say. There are different levels of support. These include holding the hand or finger, elbow support or just touching the person’s shoulder.

**What are the problems?**

There are a number of concerns about FC. These include:

·         The facilitator needs to be able to guess when the person wants to move. There is a chance that the facilitator might be responding to their own beliefs about what the person wants to type.

·         FC uses a lot of physical support to make the movements, even if the person has good movement for other activities.

·         There is not good evidence about movement difficulties (such as apraxia) in people on the spectrum.

·         Facilitators need to have a strong belief in FC and not all people can learn to be facilitators.

·         The writing done by some people using FC is surprisingly high level, even when it hasn’t been taught. Sometimes, the language and words used are unusual for a person of that age.

·         Sometimes, people using FC choose not to communicate with people close to them, even though they cooperate and interact with them in other ways. It is not clear why this happens.

·         We don’t know why facilitation is needed when some communication devices can now be used by moving a single muscle.

·         Even though there have been many reports about abuse made by people using FC, none have been found to be true.

**What does the research say?**

Reports that are just based on case studies or descriptions of FC often report good outcomes. Some studies have used more scientific methods. These studies have mostly found that the FC is being influenced, either accidently or on purpose, by the facilitator. Some studies have found that individuals using FC can only answer questions when the facilitator hears the question. If the facilitator doesn’t hear the question, or hears a different question, they produce the wrong answer. Some studies have shown that the facilitator might not realise that they are influencing the person they are trying to help.

A small number of people on the spectrum who start communicating using FC go on to use typing without help. It would be helpful to know more about these people and how they became independent.

**In summary**

FC is a controversial intervention that is not supported by research.

The American Psychological Association (APA) says:

“facilitated communication is a controversial and unproved communicative procedure with no scientifically demonstrated support for its efficacy”.

 Families should talk with a speech pathologist who specialises in complex communication needs for more information.

# Optimised Debunking Training Materials

This is a study run by a team of independent researchers from across the country. The team comprises both academics and clinicians working in the field.

It is led by Dr Jessica Paynter, a psychologist and lecturer from Griffith University.

 
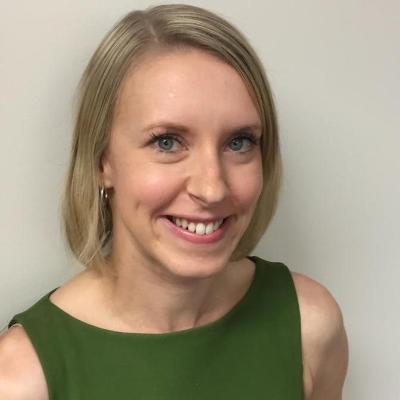


Together, we are driven by our aspiration to provide the best possible care for people living with autism spectrum disorder (autism from hereon) in Australia. We work with educational and health-care professionals like you to assist them in their decision making when it comes to selecting optimal intervention and support services.

First, we would like to thank you for your participation – we value your commitment to quality care. We hope you find participation helpful!

In the following, we will review evidence for some specific autism interventions. In general, among clinicians working with people with autism, there is a strong agreement that intervention recommendations should be based on the best available evidence.

Unfortunately, it is not always clear to clients and practitioners what the evidence base for an intervention really is. For example, sometimes intervention decisions are made based on anecdotal and/or unreliable evidence. A practitioner might think that a certain autism intervention is effective based on a recent experience:


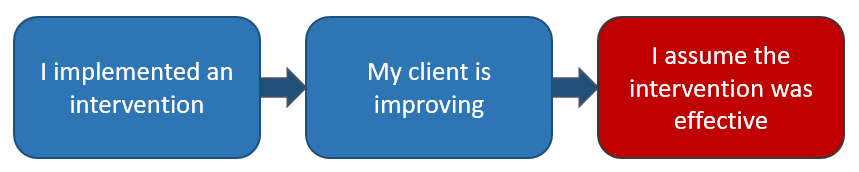


However, this conclusion can be based on what is known as an ‘illusion of causality’. If there are events that happen at the same time, or close together, people tend to link one event with the other. That is, people often see causal connections even where there are none; for example, if you have a cold and you take medication, your eventual recovery may be caused by the medication, but it may just be due to the body's natural immune response.

Inferring causal connections from day-to-day experience can be particularly difficult when working with children with autism because often an integrated approach is used, combining on average seven different interventions.[1] This makes it difficult to know what exactly is causing any improvements observed.

This is why the effectiveness of each intervention needs to be assessed in large, well-designed studies. The gold standard are randomised control trials, where people are randomly assigned to intervention and control conditions. To assess effectiveness, one needs to know: How many clients improved after the intervention (and by how much)? How many clients improved without the intervention (and by how much)? A rigorous, well-designed study can thus determine whether or not improvements are actually caused by an intervention.

[1] Green, V. A., Keenan, A., Pituch, J. I., Chois, A., O’Reilly, M., & Sigafoos, J. (2006). Internet survey of treatments used by parents of children with autism. Research in Developmental Disabilities, 27, 70-84.

For some interventions that are still commonly recommended and applied in practice, research has shown that there is no causal connection between the interventions and symptom improvements.

To understand why, in some cases, ineffective interventions are still being recommended and used, it is important to keep in mind that parents of children with autism, as well as professionals working with them, are eager to help the children – so much so that some parents and professionals are often willing to give anything a go. This situation is also fuelled by misinformation spread by online media and entertainment sources. However, as practitioners, we have an obligation to ensure that (1) resources are not wasted but instead used for the most promising interventions, (2) parents are not given false hope, and (3) clients are not exposed to avoidable harms.

Therefore, it is important to communicate the evidence clearly and to select intervention practices for our clients that are based on the best available evidence. There is a strong agreement among health-care professionals that only interventions that work should be recommended. To facilitate this, we will now review the evidence for specific autism interventions. We will first give an example of an effective intervention, and then we will debunk some intervention myths in the field of autism.


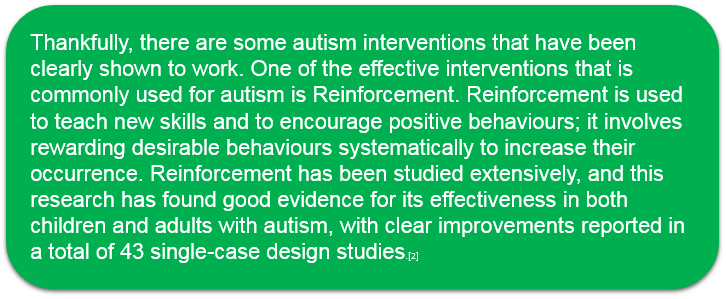


[2] Wong et al. (2015). Evidence-based practices for children, youth, and young adults with autism spectrum disorder. A comprehensive review. Journal of Autism and Developmental Disorders. 45(7), 1951-1966.

The evidence supporting Reinforcement is illustrated in the figure below - it shows that reinforcement has been found effective in the overwhelming majority of studies:


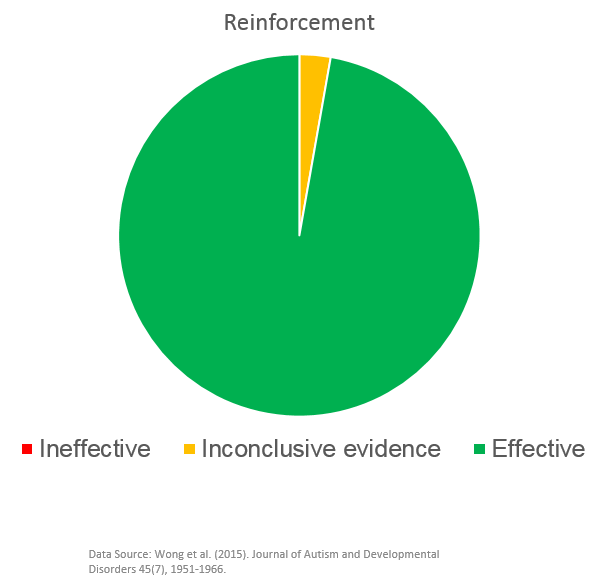


We will now turn to interventions that research has found to be **ineffective**.**Specific interventions that are known to be ineffective –
Facilitated Communication (FC)**

**Myth:** Clients with autism benefit from a practice known as Facilitated Communication (FC), also called “Rapid Prompting”, “Supported Typing”, “Informative Pointing”, “Progressive Kinesthetic Feedback”, or “Written Output Communication Enhancement”.

FC as an intervention for autism is based on the idea that autism reflects primarily a physical inability to communicate rather than a neurodevelopmental disability. FC involves providing an alphabet board (or a board with words or pictures) to the person with autism.  A “facilitator” then makes physical contact with the hand, wrist, or arm of the person with autism, while that person touches the symbols (letters, words, or pictures). FC may be particularly appealing because it conceptualises individuals with autism as unimpaired minds locked in malfunctioning bodies. However, research has shown that FC taps into the beliefs and expectations of the facilitator, rather than those of the person with autism. Consequently, the messages supposedly “produced” by the client actually reflect the conscious or subconscious intentions of the facilitator.

**A large body of empirical research has shown FC to be entirely ineffective as an intervention for autism.**

**
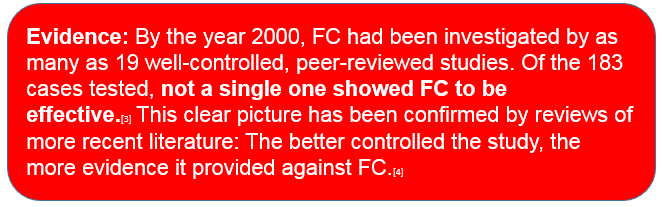
**

[3] Gorman, B. J. (1999). Facilitated communication: Rejected in science, accepted in court—A case study and analysis of the use of FC evidence under Frye and Daubert. Behavioral Sciences and the Law, 17, 517-541;  Lilienfeld, S. O. (2005). Scientifically unsupported and supported interventions for childhood psychopathology: A summary. Pediatrics, 115, 761-764.

[4] Mostert, M. P. (2010). Facilitated communication and its legitimacy—Twenty-first century developments. Exceptionality, 18, 31-41.

As a consequence, FC is now **rejected unanimously** by relevant professional organisations including Speech Pathology Australia, the Victorian Advocacy League for Individuals with Disability (VALID), the American Speech Language Hearing Association, the American Psychological Association, and even the International Society for Augmentative and Alternative Communication. Moreover, not only has FC been found ineffective, it has been shown to cause harm in some cases.[5]

[5] Sigafoos, J., & Schlosser, R. (2012). An experiential account of facilitated communication. Evidence-Based Communication Assessment and Intervention, 6, 1-2;  Boynton, J. (2012). Facilitated communication—What harm it can do: Confessions of a former facilitator. Evidence-Based Communication Assessment and Intervention, 6, 3-13.

The evidence against FC is illustrated in the figure below - No high-quality studies have found FC to be effective.


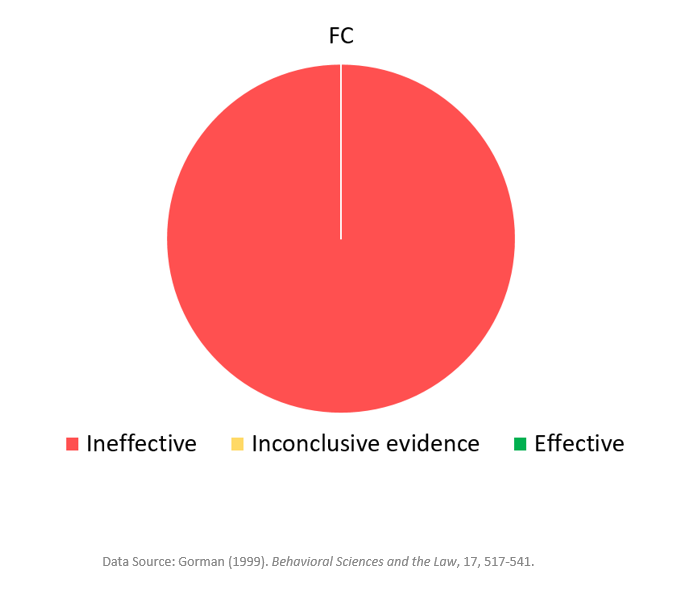


As FC has been thoroughly invalidated, an overwhelming majority of expert professionals advise against this practice.

**
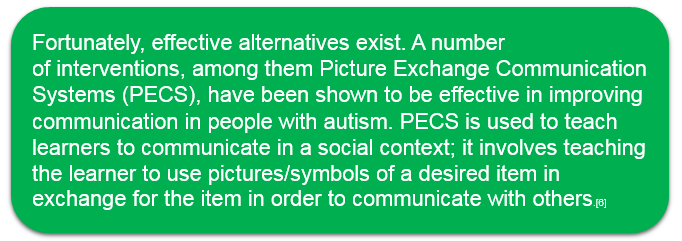
**

[6] Wong et al. (2015). Evidence-based practices for children, youth, and young adults with autism spectrum disorder. A comprehensive review. Journal of Autism and Developmental Disorders. 45(7), 1951-1966.

In the photo below: PECS being implemented at the XXXX Early Learning and Care Centre.

**[Photo from Centre]**

**Specific interventions that are known to be ineffective – Auditory Integration Training (AIT)**

**Myth:** Clients with autism benefit from a practice known as Auditory Integration Training (AIT), also called Auditory Enhancement Training or Audio-Psycho-Phonology, or Tomatis Method.

AIT as an intervention for autism is based on the hypothesis that there is an abnormal sound sensitivity in people with autism that underpins challenging behaviours and learning difficulties. Generally, AIT involves listening to electronically modified music using headphones during several daily sessions over a period of time (e.g., 10 days). AIT claims to reduce the abnormal sound sensitivity, thereby “re-educating” the hearing process. AIT may be appealing because it portrays autism as a treatable hearing problem rather than a neurodevelopmental disorder. However, there is no plausible mechanism for how AIT could have any effect on hearing or “sound sensitivity,” let alone on behavioural and learning difficulties in autism.

**High quality studies have discredited AIT as an intervention for autism.**


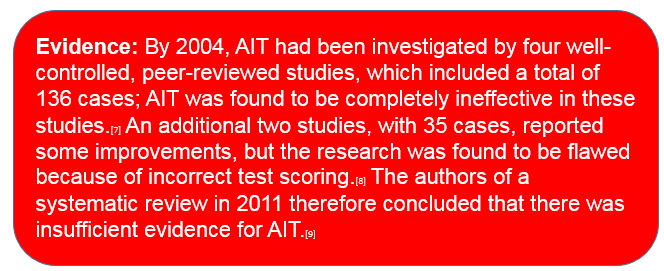


[7] Seida, J. K., Ospina, M. B., Karkhaneh, M., Hartling, L., Smith, V., & Clark, B. (2009). Systematic reviews of psychosocial interventions for autism: An umbrella review. Developmental Medicine & Child Neurology, 51, 95-104.

[8] Sinha, Y., Silove, N., Wheeler, D., & Williams, K. (2006). Auditory integration training and other sound therapies for autism spectrum disorders: A systematic review. Archives of Disease in Childhood, 91, 1018-1022.

[9] Sinha, Y., Silove, N., Hayen, A., & Williams, K. (2011). Auditory integration training and other sound therapies for autism spectrum disorders (ASD). Cochrane Database of Systematic Reviews, 12, CD003681.

On this basis, AIT is broadly rejected as an intervention for autism, and many reputable international organisations have denounced this practice as invalid, including The American Academy of Audiology, the American Speech Language Hearing Association, the Educational Audiology Association, and the American Academy of Pediatrics. Moreover, the US Food and Drug Administration has banned a range of AIT equipment, as the machine’s output levels exceeded safe limits, potentially causing distress and hearing loss.

The evidence against AIT is illustrated in the figure below - No high-quality studies have found AIT to be effective.


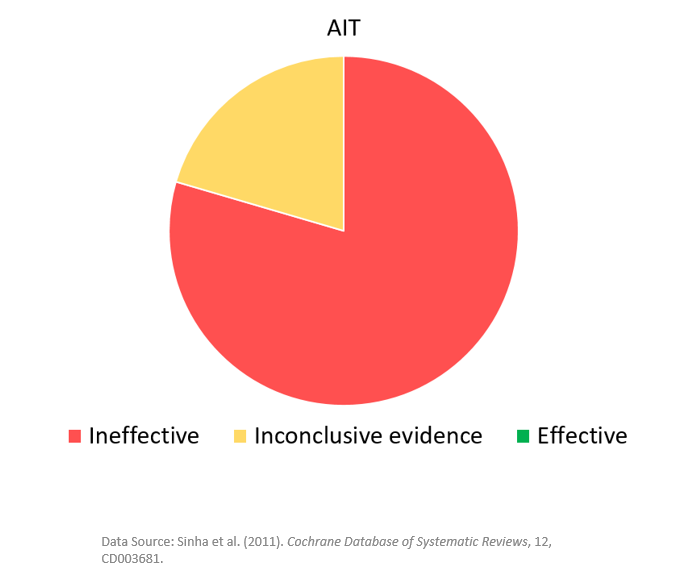


Based on the lack of evidence for AIT, an overwhelming majority of expert professionals advise against this practice.


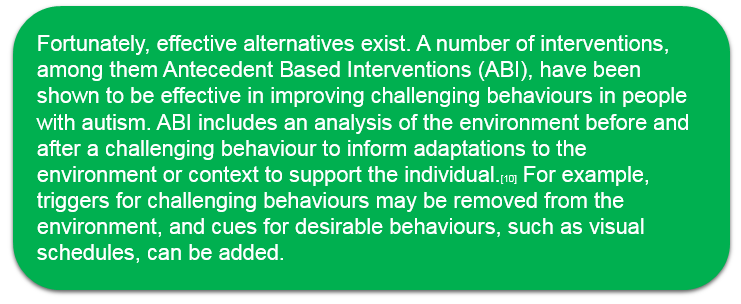


[10] Wong et al. (2015). Evidence-based practices for children, youth, and young adults with autism spectrum disorder. A comprehensive review. Journal of Autism and Developmental Disorders. 45(7), 1951-1966.

In the photo below: ABI (addition of a visual schedule to support understanding of the routine and varying access to desirable/less desirable activities) being implemented at the XXXX Autism Specific Early Learning and Care Centre.

**[Photo from Centre]**

**Specific interventions that are known to be ineffective - Special Diets**

**Myth:** Clients with autism benefit from special dietary interventions (e.g., gluten-free and casein-free [GFCF] diets).

Using GFCF diets as an intervention for autism is based on a theory that individuals with autism have increased gut permeability (a measure of how easily substances can leak through the gut walls), which leads to excessive levels of opioid peptides (peptides are chemical molecules similar to proteins that occur naturally in the human body and in food) reaching the brain. This intervention eliminates all foods containing gluten (grains such as wheat and rye) and casein (dairy products such as milk and cheese). The rationale is that eliminating certain foods producing opioid peptides can reduce autism symptoms and improve a child’s overall functioning and well-being. The appeal of this view is that it portrays autism as a problem with digestion rather than a neurodevelopmental disorder. However, there is no good evidence for increased gut permeability or elevated levels of opioid peptides in autism, so there is no plausible mechanism for a restrictive diet to reduce behavioural and learning difficulties in autism.

**Accordingly, there is inadequate evidence for the effectiveness of special diets in the treatment of core autism symptoms.**


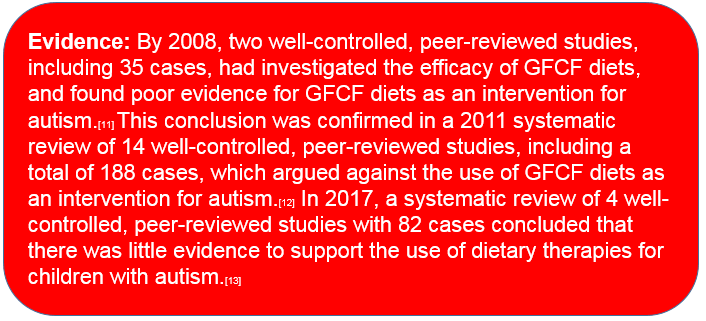


[11] Millward, C., Ferriter, M., Calver, S. J., & Connell-Jones, G. G. (2008). Gluten- and casein-free diets for autistic spectrum disorder. Cochrane Database of Systematic Reviews, 2, CD003498.

[12] Mulloy, A., Lang, R., O’Reilly, M., Sigafoos, J., Lancioni, G., & Rispoli, M. (2010). Gluten-free and casein-free diets in the treatment of autism spectrum disorders: A systematic review. Research in Autism Spectrum Disorders, 4, 328-339.

[13] Sathe, N., Andrews, J. C., McPheeters, M. L., Warren, Z. E. (2017). Nutritional and dietary interventions for autism spectrum disorder: A systematic review. Pediatrics, 139, e2017034.

As a consequence, GFCF diets are currently rejected by relevant professional organisations including The National Institute for Health and Care Excellence (NICE) and the American Academy of Pediatrics. Furthermore, special diets have been associated with adverse financial, social, and health side-effects.[14]

[14] Mulloy, A., Lang, R., O’Reilly, M., Sigafoos, J., Lancioni, G., & Rispoli, M. (2010). Gluten-free and casein-free diets in the treatment of autism spectrum disorders: A systematic review. Research in Autism Spectrum Disorders, 4, 328-339.

The evidence against special diets is illustrated in the figure below - No high-quality studies have found GFCF diets to be effective.


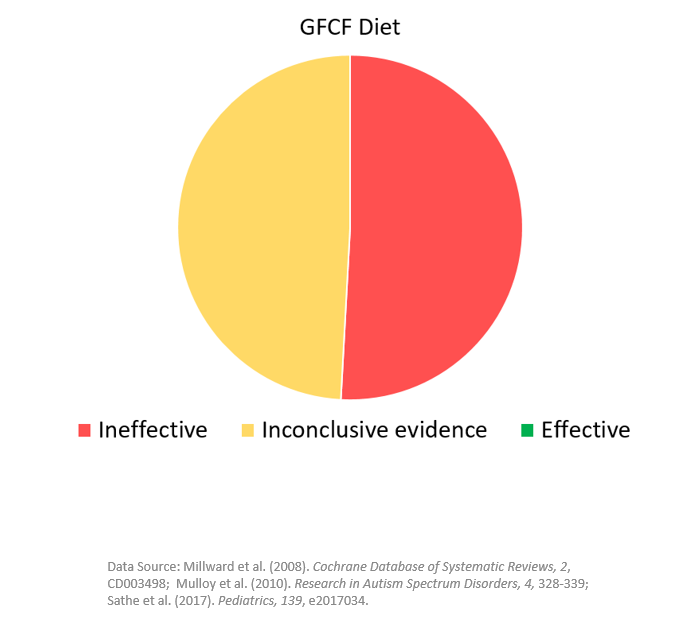


As special diets are not supported by research, an overwhelming majority of expert professionals advise against this practice as an intervention for autism.[15]

[15] Of course, special diets are useful in rare cases of documented food allergies or intolerances, such as celiac disease, which affects approx. 1% of the population (Mulloy et al., 2010).


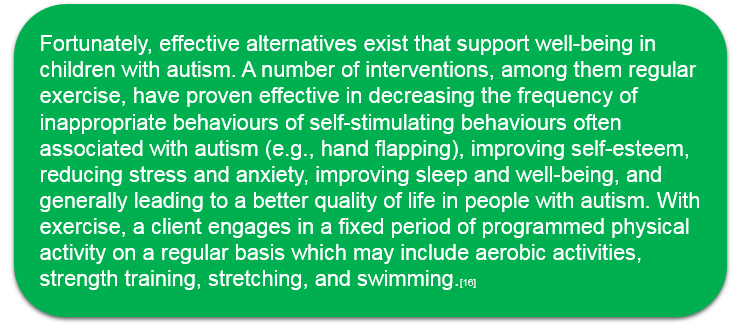


[16] Wong et al. (2015). Evidence-based practices for children, youth, and young adults with autism spectrum disorder. A comprehensive review. Journal of Autism and Developmental Disorders. 45(7), 1951-1966.

In the photo below: Exercise being implemented at the XXXX Autism Specific Early Learning and Care Centre.

**[Photo from Centre]**

# **Intervention Practices Questions**

On the following pages we are going to ask you about six different Autism intervention practices. Please read the description of each of the practices and answer the questions to the best of your ability based on your personal experience working in early intervention with children with ASD.

The questions will regard:

·        The evidence-base

·        Your clinical experience

·        Your knowledge of the practice

·        Your current use of the practice

·        Your intended future use of the practice

·        Your confidence recommending the practice to parents

·        Your confidence discussing the evidence base of this practice with parents

·        Where you received information about the practice

·        How trustworthy you find this source of information

**Practice: Facilitated Communication**

This intervention involves a ‘facilitator’ making physical contact with the hand, wrist, arm, or shoulder of a person with a disability as that person touches symbols (letters, words, pictures) on an augmentative and alternative communication (AAC) system or computer keyboard. The messages produced during this process are interpreted by those providing FC to be the person’s communication.

**Research shows this practice is**

| 0 | 1 | 2 | 3 | 4 |
| --- | --- | --- | --- | --- |
| *Ineffective:*  Research shows this practice is not effective. |  |  |  | *Established:* This practice has established its efficacy in high quality research. |

**In my clinical experience, Facilitated Communication** **is**

| 0 | 1 | 2 | 3 | 4 |
| --- | --- | --- | --- | --- |
| *Not at all effective:*  This practice is not effective. |  |  |  | *Highly effective:* This practice is highly effective. |

**My knowledge of Facilitated Communication** **is**

| 0 | 1 | 2 | 3 | 4 |
| --- | --- | --- | --- | --- |
| *Very Little:*  I know nothing about this practice. |  |  |  | *To a Very Great Extent: I k*now a great deal and could instruct others on this. |

**My current use of Facilitated Communication** **is**

| 0 | 1 | 2 | 3 | 4 |
| --- | --- | --- | --- | --- |
| *Never:*  I do not use this practice. |  |  |  | *Frequently.* |

**My intended future use in my current role with children at ASELCC as well as others**

| 0 | 1 | 2 | 3 | 4 |
| --- | --- | --- | --- | --- |
| *Never:* I will not use this practice |  |  |  | *Frequently.* |

**My confidence in making recommendations about this practice to parents**

| -3 | -2 | -1 | 1 | 2 | 3 |
| --- | --- | --- | --- | --- | --- |
| I would NOT recommend this practice to parents, and I would do so with HIGH confidence. | I would NOT recommend this practice to parents, and I would do so with MODERATE confidence. | I would NOT recommend this practice to parents, and I would do so with LOW confidence. | I would recommend this practice to parents, and I would do so with LOW confidence. | I would recommend this practice to parents, and I would do so with MODERATE confidence. | I would recommend this practice to parents, and I would do so with HIGH confidence. |

**My confidence discussing the evidence base with parents**

| 0 | 1 | 2 | 3 | 4 |
| --- | --- | --- | --- | --- |
| *Not at all confident:*  I do not feel confident discussing the evidence base of this practice with parents. |  |  |  | *Very Confident:* I feel very confident discussing the evidence base of this practice with parents. |

**Facilitated Communication**

**I received information on this practice from:**

**Please select all that apply.**

|  |  |
| --- | --- |
| Parents of children with ASD |  |
| Teachers |  |
| Research literature (e.g., academic books, academic journals) |  |
| Research websites (e.g., Cochrane Reviews, National Autism Centre) |  |
| Internal professional development or training |  |
| Therapists (e.g., Speech Therapists, Psychologists, Occupational Therapists) |  |
| Workshops or other external professional development |  |
| Treatment reviews (e.g., Good Practice Guidelines) |  |
| Websites other |  |
| Other |  |
| I have never received information about this practice |  |

**How much do you trust this source of information on Facilitated Communication**

|  | 1: *Not at all trustworthy* | 2 | 3 | 4 | 5: *Very trustworthy* |
| --- | --- | --- | --- | --- | --- |
| Parents of children with ASD |  |  |  |  |  |
| Teachers |  |  |  |  |  |
| Research literature (e.g., academic books, academic journals) |  |  |  |  |  |
| Research websites (e.g., Cochrane Reviews, National Autism Centre) |  |  |  |  |  |
| Internal professional development or training |  |  |  |  |  |
| Therapists (e.g., Speech Therapists, Psychologists, Occupational Therapists) |  |  |  |  |  |
| Workshops or other external professional development |  |  |  |  |  |
| Treatment reviews (e.g., Good Practice Guidelines) |  |  |  |  |  |
| Websites other |  |  |  |  |  |
| Other |  |  |  |  |  |
| I have never received information about this practice |  |  |  |  |  |

**Practice: Auditory Integration Training**

This intervention involves the presentation of modulated sounds through headphones to retrain an individual’s auditory system with the goal of improving sensory processing of sound.

**Research shows this practice is**

| 0 | 1 | 2 | 3 | 4 |
| --- | --- | --- | --- | --- |
| *Ineffective:*  Research shows this practice is not effective. |  |  |  | *Established:* This practice has established its efficacy in high quality research. |

**In my clinical experience, Auditory Integration Training** **is**

| 0 | 1 | 2 | 3 | 4 |
| --- | --- | --- | --- | --- |
| *Not at all effective:*  This practice is not effective. |  |  |  | *Highly effective:* This practice is highly effective. |

**My knowledge of Auditory Integration Training** **is**

| 0 | 1 | 2 | 3 | 4 |
| --- | --- | --- | --- | --- |
| *Very Little:*  I know nothing about this practice. |  |  |  | *To a Very Great Extent: I k*now a great deal and could instruct others on this. |

**My current use of Auditory Integration Training** **is**

| 0 | 1 | 2 | 3 | 4 |
| --- | --- | --- | --- | --- |
| *Never:*  I do not use this practice. |  |  |  | *Frequently.* |

**My intended future use in my current role with children at ASELCC as well as others**

| 0 | 1 | 2 | 3 | 4 |
| --- | --- | --- | --- | --- |
| *Never:* I will not use this practice |  |  |  | *Frequently.* |

**My confidence in making recommendations about this practice to parents**

| -3 | -2 | -1 | 1 | 2 | 3 |
| --- | --- | --- | --- | --- | --- |
| I would NOT recommend this practice to parents, and I would do so with HIGH confidence. | I would NOT recommend this practice to parents, and I would do so with MODERATE confidence. | I would NOT recommend this practice to parents, and I would do so with LOW confidence. | I would recommend this practice to parents, and I would do so with LOW confidence. | I would recommend this practice to parents, and I would do so with MODERATE confidence. | I would recommend this practice to parents, and I would do so with HIGH confidence. |

**My confidence discussing the evidence base with parents**

| 0 | 1 | 2 | 3 | 4 |
| --- | --- | --- | --- | --- |
| *Not at all confident:*  I do not feel confident discussing the evidence base of this practice with parents. |  |  |  | *Very Confident:* I feel very confident discussing the evidence base of this practice with parents. |

**Auditory Integration Training**

**I received information on this practice from:**

**Please select all that apply.**

|  |  |
| --- | --- |
| Parents of children with ASD |  |
| Teachers |  |
| Research literature (e.g., academic books, academic journals) |  |
| Research websites (e.g., Cochrane Reviews, National Autism Centre) |  |
| Internal professional development or training |  |
| Therapists (e.g., Speech Therapists, Psychologists, Occupational Therapists) |  |
| Workshops or other external professional development |  |
| Treatment reviews (e.g., Good Practice Guidelines) |  |
| Websites other |  |
| Other |  |
| I have never received information about this practice |  |

**How much do you trust this source of information on Auditory Integration Training**

|  | 1: *Not at all trustworthy* | 2 | 3 | 4 | 5: *Very trustworthy* |
| --- | --- | --- | --- | --- | --- |
| Parents of children with ASD |  |  |  |  |  |
| Teachers |  |  |  |  |  |
| Research literature (e.g., academic books, academic journals) |  |  |  |  |  |
| Research websites (e.g., Cochrane Reviews, National Autism Centre) |  |  |  |  |  |
| Internal professional development or training |  |  |  |  |  |
| Therapists (e.g., Speech Therapists, Psychologists, Occupational Therapists) |  |  |  |  |  |
| Workshops or other external professional development |  |  |  |  |  |
| Treatment reviews (e.g., Good Practice Guidelines) |  |  |  |  |  |
| Websites other |  |  |  |  |  |
| Other |  |  |  |  |  |
| I have never received information about this practice |  |  |  |  |  |

**Practice: Antecedent Based Intervention**

This intervention involves anticipating and arranging events or circumstances that come before a challenging behaviour with the aim of reducing or preventing the behaviour. This intervention is sometimes used as part of the Positive Behaviour Support model which includes strengthening positive behaviours as well as anticipating and preventing challenging behaviours.

**Research shows this practice is**

| 0 | 1 | 2 | 3 | 4 |
| --- | --- | --- | --- | --- |
| *Ineffective:*  Research shows this practice is not effective. |  |  |  | *Established:* This practice has established its efficacy in high quality research. |

**In my clinical experience, Antecedent Based Intervention** **is**

| 0 | 1 | 2 | 3 | 4 |
| --- | --- | --- | --- | --- |
| *Not at all effective:*  This practice is not effective. |  |  |  | *Highly effective:* This practice is highly effective. |

**My knowledge of Antecedent Based Intervention is**

| 0 | 1 | 2 | 3 | 4 |
| --- | --- | --- | --- | --- |
| *Very Little:*  I know nothing about this practice. |  |  |  | *To a Very Great Extent: I k*now a great deal and could instruct others on this. |

**My current use of Antecedent Based Intervention** **is**

| 0 | 1 | 2 | 3 | 4 |
| --- | --- | --- | --- | --- |
| *Never:*  I do not use this practice. |  |  |  | *Frequently.* |

**My intended future use in my current role with children at ASELCC as well as others**

| 0 | 1 | 2 | 3 | 4 |
| --- | --- | --- | --- | --- |
| *Never:* I will not use this practice |  |  |  | *Frequently.* |

**My confidence in making recommendations about this practice to parents**

| -3 | -2 | -1 | 1 | 2 | 3 |
| --- | --- | --- | --- | --- | --- |
| I would NOT recommend this practice to parents, and I would do so with HIGH confidence. | I would NOT recommend this practice to parents, and I would do so with MODERATE confidence. | I would NOT recommend this practice to parents, and I would do so with LOW confidence. | I would recommend this practice to parents, and I would do so with LOW confidence. | I would recommend this practice to parents, and I would do so with MODERATE confidence. | I would recommend this practice to parents, and I would do so with HIGH confidence. |

**My confidence discussing the evidence base with parents**

| 0 | 1 | 2 | 3 | 4 |
| --- | --- | --- | --- | --- |
| *Not at all confident:*  I do not feel confident discussing the evidence base of this practice with parents. |  |  |  | *Very Confident:* I feel very confident discussing the evidence base of this practice with parents. |

**Antecedent Based Intervention**

**I received information on this practice from:**

**Please select all that apply.**

|  |  |
| --- | --- |
| Parents of children with ASD |  |
| Teachers |  |
| Research literature (e.g., academic books, academic journals) |  |
| Research websites (e.g., Cochrane Reviews, National Autism Centre) |  |
| Internal professional development or training |  |
| Therapists (e.g., Speech Therapists, Psychologists, Occupational Therapists) |  |
| Workshops or other external professional development |  |
| Treatment reviews (e.g., Good Practice Guidelines) |  |
| Websites other |  |
| Other |  |
| I have never received information about this practice |  |

**How much do you trust this source of information on Antecedent Based Intervention**

|  | 1: *Not at all trustworthy* | 2 | 3 | 4 | 5: *Very trustworthy* |
| --- | --- | --- | --- | --- | --- |
| Parents of children with ASD |  |  |  |  |  |
| Teachers |  |  |  |  |  |
| Research literature (e.g., academic books, academic journals) |  |  |  |  |  |
| Research websites (e.g., Cochrane Reviews, National Autism Centre) |  |  |  |  |  |
| Internal professional development or training |  |  |  |  |  |
| Therapists (e.g., Speech Therapists, Psychologists, Occupational Therapists) |  |  |  |  |  |
| Workshops or other external professional development |  |  |  |  |  |
| Treatment reviews (e.g., Good Practice Guidelines) |  |  |  |  |  |
| Websites other |  |  |  |  |  |
| Other |  |  |  |  |  |
| I have never received information about this practice |  |  |  |  |  |

**Practice: Exercise**

These interventions involve an increase in physical exertion as a means of reducing problem behaviours or increasing appropriate behaviour.

**Research shows this practice is**

| 0 | 1 | 2 | 3 | 4 |
| --- | --- | --- | --- | --- |
| *Ineffective:*  Research shows this practice is not effective. |  |  |  | *Established:* This practice has established its efficacy in high quality research. |

**In my clinical experience, exercise as an intervention for ASD is**

| 0 | 1 | 2 | 3 | 4 |
| --- | --- | --- | --- | --- |
| *Not at all effective:*  This practice is not effective. |  |  |  | *Highly effective:* This practice is highly effective. |

**My knowledge of exercise as an intervention for ASD is**

| 0 | 1 | 2 | 3 | 4 |
| --- | --- | --- | --- | --- |
| *Very Little:*  I know nothing about this practice. |  |  |  | *To a Very Great Extent: I k*now a great deal and could instruct others on this. |

**My current use of exercise as an intervention for ASD is**

| 0 | 1 | 2 | 3 | 4 |
| --- | --- | --- | --- | --- |
| *Never:*  I do not use this practice. |  |  |  | *Frequently.* |

**My intended future use in my current role with children at ASELCC as well as others**

| 0 | 1 | 2 | 3 | 4 |
| --- | --- | --- | --- | --- |
| *Never:* I will not use this practice |  |  |  | *Frequently.* |

**My confidence in making recommendations about this practice to parents**

| -3 | -2 | -1 | 1 | 2 | 3 |
| --- | --- | --- | --- | --- | --- |
| I would NOT recommend this practice to parents, and I would do so with HIGH confidence. | I would NOT recommend this practice to parents, and I would do so with MODERATE confidence. | I would NOT recommend this practice to parents, and I would do so with LOW confidence. | I would recommend this practice to parents, and I would do so with LOW confidence. | I would recommend this practice to parents, and I would do so with MODERATE confidence. | I would recommend this practice to parents, and I would do so with HIGH confidence. |

**My confidence discussing the evidence base with parents**

| 0 | 1 | 2 | 3 | 4 |
| --- | --- | --- | --- | --- |
| *Not at all confident:*  I do not feel confident discussing the evidence base of this practice with parents. |  |  |  | *Very Confident:* I feel very confident discussing the evidence base of this practice with parents. |

**Exercise**

**I received information on this practice from:**

**Please select all that apply.**

|  |  |
| --- | --- |
| Parents of children with ASD |  |
| Teachers |  |
| Research literature (e.g., academic books, academic journals) |  |
| Research websites (e.g., Cochrane Reviews, National Autism Centre) |  |
| Internal professional development or training |  |
| Therapists (e.g., Speech Therapists, Psychologists, Occupational Therapists) |  |
| Workshops or other external professional development |  |
| Treatment reviews (e.g., Good Practice Guidelines) |  |
| Websites other |  |
| Other |  |
| I have never received information about this practice |  |

**How much do you trust this source of information on Exercise**

|  | 1: *Not at all trustworthy* | 2 | 3 | 4 | 5: *Very trustworthy* |
| --- | --- | --- | --- | --- | --- |
| Parents of children with ASD |  |  |  |  |  |
| Teachers |  |  |  |  |  |
| Research literature (e.g., academic books, academic journals) |  |  |  |  |  |
| Research websites (e.g., Cochrane Reviews, National Autism Centre) |  |  |  |  |  |
| Internal professional development or training |  |  |  |  |  |
| Therapists (e.g., Speech Therapists, Psychologists, Occupational Therapists) |  |  |  |  |  |
| Workshops or other external professional development |  |  |  |  |  |
| Treatment reviews (e.g., Good Practice Guidelines) |  |  |  |  |  |
| Websites other |  |  |  |  |  |
| Other |  |  |  |  |  |
| I have never received information about this practice |  |  |  |  |  |

**Practice: Special Diets (e.g. Gluten free diet, casein diet)**

These interventions involve dietary intervention, such as eliminating gluten (e.g., wheat, barley, rye) or casein (e.g., dairy products) with the goal of improving child general functioning and/or autism symptoms.

**Research shows this practice is**

| 0 | 1 | 2 | 3 | 4 |
| --- | --- | --- | --- | --- |
| *Ineffective:*  Research shows this practice is not effective. |  |  |  | *Established:* This practice has established its efficacy in high quality research. |

**In my clinical experience, special diets as an intervention for ASD is**

| 0 | 1 | 2 | 3 | 4 |
| --- | --- | --- | --- | --- |
| *Not at all effective:*  This practice is not effective. |  |  |  | *Highly effective:* This practice is highly effective. |

**My knowledge of special diets as an intervention for ASD is**

| 0 | 1 | 2 | 3 | 4 |
| --- | --- | --- | --- | --- |
| *Very Little:*  I know nothing about this practice. |  |  |  | *To a Very Great Extent: I k*now a great deal and could instruct others on this. |

**My current use of special diets as an intervention for ASD is**

| 0 | 1 | 2 | 3 | 4 |
| --- | --- | --- | --- | --- |
| *Never:*  I do not use this practice. |  |  |  | *Frequently.* |

**My intended future use in my current role with children at ASELCC as well as others**

| 0 | 1 | 2 | 3 | 4 |
| --- | --- | --- | --- | --- |
| *Never:* I will not use this practice |  |  |  | *Frequently.* |

**My confidence in making recommendations about this practice to parents**

| -3 | -2 | -1 | 1 | 2 | 3 |
| --- | --- | --- | --- | --- | --- |
| I would NOT recommend this practice to parents, and I would do so with HIGH confidence. | I would NOT recommend this practice to parents, and I would do so with MODERATE confidence. | I would NOT recommend this practice to parents, and I would do so with LOW confidence. | I would recommend this practice to parents, and I would do so with LOW confidence. | I would recommend this practice to parents, and I would do so with MODERATE confidence. | I would recommend this practice to parents, and I would do so with HIGH confidence. |

**My confidence discussing the evidence base with parents**

| 0 | 1 | 2 | 3 | 4 |
| --- | --- | --- | --- | --- |
| *Not at all confident:*  I do not feel confident discussing the evidence base of this practice with parents. |  |  |  | *Very Confident:* I feel very confident discussing the evidence base of this practice with parents. |

**Special Diets (e.g. Gluten free diet, casein diet)**

**I received information on this practice from:**

**Please select all that apply.**

|  |  |
| --- | --- |
| Parents of children with ASD |  |
| Teachers |  |
| Research literature (e.g., academic books, academic journals) |  |
| Research websites (e.g., Cochrane Reviews, National Autism Centre) |  |
| Internal professional development or training |  |
| Therapists (e.g., Speech Therapists, Psychologists, Occupational Therapists) |  |
| Workshops or other external professional development |  |
| Treatment reviews (e.g., Good Practice Guidelines) |  |
| Websites other |  |
| Other |  |
| I have never received information about this practice |  |

**How much do you trust this source of information on Special Diets (e.g. Gluten free diet, casein diet)**

|  | 1: *Not at all trustworthy* | 2 | 3 | 4 | 5: *Very trustworthy* |
| --- | --- | --- | --- | --- | --- |
| Parents of children with ASD |  |  |  |  |  |
| Teachers |  |  |  |  |  |
| Research literature (e.g., academic books, academic journals) |  |  |  |  |  |
| Research websites (e.g., Cochrane Reviews, National Autism Centre) |  |  |  |  |  |
| Internal professional development or training |  |  |  |  |  |
| Therapists (e.g., Speech Therapists, Psychologists, Occupational Therapists) |  |  |  |  |  |
| Workshops or other external professional development |  |  |  |  |  |
| Treatment reviews (e.g., Good Practice Guidelines) |  |  |  |  |  |
| Websites other |  |  |  |  |  |
| Other |  |  |  |  |  |
| I have never received information about this practice |  |  |  |  |  |

**Practice: Picture Exchange Communication System (PECS)**

This intervention involves teaching children to exchange pictures with others to communicate.

**Research shows this practice is**

| 0 | 1 | 2 | 3 | 4 |
| --- | --- | --- | --- | --- |
| *Ineffective:*  Research shows this practice is not effective. |  |  |  | *Established:* This practice has established its efficacy in high quality research. |

**In my clinical experience, Picture Exchange Communication System is**

| 0 | 1 | 2 | 3 | 4 |
| --- | --- | --- | --- | --- |
| *Not at all effective:*  This practice is not effective. |  |  |  | *Highly effective:* This practice is highly effective. |

**My knowledge of Picture Exchange Communication System is**

| 0 | 1 | 2 | 3 | 4 |
| --- | --- | --- | --- | --- |
| *Very Little:*  I know nothing about this practice. |  |  |  | *To a Very Great Extent: I k*now a great deal and could instruct others on this. |

**My current use of Picture Exchange Communication System is**

| 0 | 1 | 2 | 3 | 4 |
| --- | --- | --- | --- | --- |
| *Never:*  I do not use this practice. |  |  |  | *Frequently.* |

**My intended future use in my current role with children at ASELCC as well as others**

| 0 | 1 | 2 | 3 | 4 |
| --- | --- | --- | --- | --- |
| *Never:* I will not use this practice |  |  |  | *Frequently.* |

**My confidence in making recommendations about this practice to parents**

| -3 | -2 | -1 | 1 | 2 | 3 |
| --- | --- | --- | --- | --- | --- |
| I would NOT recommend this practice to parents, and I would do so with HIGH confidence. | I would NOT recommend this practice to parents, and I would do so with MODERATE confidence. | I would NOT recommend this practice to parents, and I would do so with LOW confidence. | I would recommend this practice to parents, and I would do so with LOW confidence. | I would recommend this practice to parents, and I would do so with MODERATE confidence. | I would recommend this practice to parents, and I would do so with HIGH confidence. |

**My confidence discussing the evidence base with parents**

| 0 | 1 | 2 | 3 | 4 |
| --- | --- | --- | --- | --- |
| *Not at all confident:*  I do not feel confident discussing the evidence base of this practice with parents. |  |  |  | *Very Confident:* I feel very confident discussing the evidence base of this practice with parents. |

**Picture Exchange Communication System (PECS)**

**I received information on this practice from:**

**Please select all that apply.**

|  |  |
| --- | --- |
| Parents of children with ASD |  |
| Teachers |  |
| Research literature (e.g., academic books, academic journals) |  |
| Research websites (e.g., Cochrane Reviews, National Autism Centre) |  |
| Internal professional development or training |  |
| Therapists (e.g., Speech Therapists, Psychologists, Occupational Therapists) |  |
| Workshops or other external professional development |  |
| Treatment reviews (e.g., Good Practice Guidelines) |  |
| Websites other |  |
| Other |  |
| I have never received information about this practice |  |

**How much do you trust this source of information on Picture Exchange Communication System (PECS)**

|  | 1: *Not at all trustworthy* | 2 | 3 | 4 | 5: *Very trustworthy* |
| --- | --- | --- | --- | --- | --- |
| Parents of children with ASD |  |  |  |  |  |
| Teachers |  |  |  |  |  |
| Research literature (e.g., academic books, academic journals) |  |  |  |  |  |
| Research websites (e.g., Cochrane Reviews, National Autism Centre) |  |  |  |  |  |
| Internal professional development or training |  |  |  |  |  |
| Therapists (e.g., Speech Therapists, Psychologists, Occupational Therapists) |  |  |  |  |  |
| Workshops or other external professional development |  |  |  |  |  |
| Treatment reviews (e.g., Good Practice Guidelines) |  |  |  |  |  |
| Websites other |  |  |  |  |  |
| Other |  |  |  |  |  |
| I have never received information about this practice |  |  |  |  |  |

# Social Validity

**How I Found the Online Training**

**The online training was an acceptable way to improve my knowledge of autism spectrum disorder interventions**

| 1 | 2 | 3 | 4 | 5 | 6 |
| --- | --- | --- | --- | --- | --- |
| *Strongly disagree* |  |  |  |  | *Strongly Agree* |

**Most practitioners would find the online training appropriate**

| 1 | 2 | 3 | 4 | 5 | 6 |
| --- | --- | --- | --- | --- | --- |
| *Strongly disagree* |  |  |  |  | *Strongly Agree* |

**I would recommend the online training to help practitioners learn more about specific autism intervention practices**

| 1 | 2 | 3 | 4 | 5 | 6 |
| --- | --- | --- | --- | --- | --- |
| *Strongly disagree* |  |  |  |  | *Strongly Agree* |

**The online training increased my confidence in identifying effective interventions**

| 1 | 2 | 3 | 4 | 5 | 6 |
| --- | --- | --- | --- | --- | --- |
| *Strongly disagree* |  |  |  |  | *Strongly Agree* |

**The online training is appropriate to use to upskill practitioners working with children with autism**

| 1 | 2 | 3 | 4 | 5 | 6 |
| --- | --- | --- | --- | --- | --- |
| *Strongly disagree* |  |  |  |  | *Strongly Agree* |

**Overall, the online training was beneficial**

| 1 | 2 | 3 | 4 | 5 | 6 |
| --- | --- | --- | --- | --- | --- |
| *Strongly disagree* |  |  |  |  | *Strongly Agree* |

# **Manipulation Check**

**The training materials I just viewed included charts (e.g., charts or pie charts) showing the evidence relating to the interventions being discussed**

| 1 | 2 | 3 | 4 | 5 |
| --- | --- | --- | --- | --- |
| *Strongly disagree* |  |  |  | *Strongly Agree* |

**The training materials gave me alternative options for the three ineffective interventions discussed**

| 1 | 2 | 3 | 4 | 5 |
| --- | --- | --- | --- | --- |
| *Strongly disagree* |  |  |  | *Strongly Agree* |

**The training materials reported that professional organisations advise AGAINST the three ineffective interventions discussed**

| 1 | 2 | 3 | 4 | 5 |
| --- | --- | --- | --- | --- |
| *Strongly disagree* |  |  |  | *Strongly Agree* |
