# Supplementary material for: Evaluation of a template for countering misinformation—Real-world Autism treatment myth debunking
Source: PLoS One. 2019 Jan 30;14(1):e0210746. doi: 10.1371/journal.pone.0210746 (PMC6353548; doi:10.1371/journal.pone.0210746)
Supplement: S2 File — (DOCX) [file pone.0210746.s002.docx]

**Data Screening**

Missing values analysis showed 23% missing data across demographics and key variables, which were missing at random; Little’s MCAR Chi-square (85) = 78.48, *p* = .68, *ns.* As is acceptable under these conditions (e.g., Tabachnick & Fidell, 2007), data were deleted listwise, leaving a remaining sample of 47 participants for the main analyses (2 × 2 ANOVAs) and 42 for the follow-up analyses. Data were screened for meeting assumptions of *t*-tests, ANOVA, and correlations for each analysis. Outliers were detected using boxplots and studentized residuals (using *z* = +/- 3) for the manipulation check for question 1 (control, *n* = 5; debunking, *n* = 2), question 2 (debunking, *n* = 1), and question 3 (debunking, *n* = 1). Outliers were also detected for the ANOVA for ESTs (*n* = 1) and one outlier was detected on the social-validity measure. Each of the outlying data points appeared to be a true response (responses assessed for data entry errors and patterned responding), and analyses were thus run with and without these outliers. Outliers did not substantively impact on the pattern of results or effect sizes obtained, and were thus retained for analyses. Data were also screened for normality (standardized skew and kurtosis, QQ plots), homogeneity of variances (Levene’s tests *p* > .05) and covariances (all Box’s tests *p*> .001), and sphericity (using Machly’s test *p* > .05). Homogeneity of variances was violated for two *t*-tests only (Q1 and Q2 of the manipulation check); Welch’s *t*-test was conducted for these comparisons.

**Reference**

Tabachnick, B. G., & Fidell, L. S. (2007). Using multivariate statistics (5th ed.): Boston, MA:

Bacon/Pearson Education.
